# Hypofractionated radiotherapy in children with diffuse intrinsic pontine glioma

**DOI:** 10.1111/ped.14070

**Published:** 2020-01-29

**Authors:** Akiko Hayashi, Eiko Ito, Motoko Omura, Noriko Aida, Mio Tanaka, Yukichi Tanaka, Hironobu Sato, Naoyuki Miyagawa, Tomoko Yokosuka, Fuminori Iwasaki, Satoshi Hamanoue, Hiroaki Goto

**Affiliations:** ^1^ Division of Hematology/Oncology, Department of Pediatrics Kanagawa Children's Medical Center Yokohama Japan; ^2^ Department of Radiology Kanagawa Children's Medical Center Yokohama Japan; ^3^ Department of Pathology Kanagawa Children's Medical Center Yokohama Japan; ^4^ Department of Neurosurgery Kanagawa Children's Medical Center Yokohama Japan

**Keywords:** brain stem neoplasm, child, dose hypofractionation, glioma, re‐irradiation

## Abstract

**Background:**

Overall survival (OS) of patients with diffuse intrinsic pontine glioma (DIPG) is poor, with radiation therapy (RT) the only intervention that transiently delays tumor progression. Hypofractionated RT and re‐irradiation at first progression have gained popularity in improving the quality of life of such patients.

**Methods:**

We performed a retrospective review of children with DIPG treated at Kanagawa Children's Medical Center from 2000 to 2018.

**Results:**

A total of 24 cases were reviewed. Median age at diagnosis was 6.3 years (1.6–14.0). Twenty patients received RT only once. Thirteen patients received conventionally fractionated RT, and seven patients received hypofractionated RT as up‐front RT. Severe toxicities were not observed in patients who received hypofractionated RT. Median OS and time to progression were similar between conventionally fractionated and hypofractionated RT groups.(9.7 [95% confidence interval(CI): 7.1‐11.2] versus 11.0[95% CI: 5.2‐13.6] months, *P* = 0.60; 4.2[95% CI: 1.8‐8.3] versus 7.1 [95% CI:4.5‐8.7] months, *P* = 0.38). Four patients received re‐irradiation at first progression and all patients showed transient neurological improvement and survival more than a year after diagnosis. A 4‐year‐old boy was re‐irradiated 5‐and‐a‐half months after the first re‐irradiation; following transient neurological improvement. He survived a further 5 months.

**Conclusion:**

Hypofractionated RT for children with newly diagnosed DIPG is well tolerated and feasible from the viewpoint of reducing a patient's burden of treatment. Re‐irradiation at first progression is suggested to be beneficial.

Diffuse intrinsic pontine glioma (DIPG) is a malignant brain stem tumor for which curative therapy is unavailable and the median survival is <1 year.^1^ Radiation therapy (RT) is the only effective intervention that confers a short period of clinical improvement and survival benefit. To date, no chemotherapeutic strategy has been shown to improve overall survival in children with DIPG. The current standard of treatment consists of conventionally fractionated RT to a range of 50.4–59.4 Gy in 28–33 fractions of 1.8 Gy daily, over 6 weeks, followed by best supportive care.^2^ Recently, some institutions have employed hypofractionated RT to decrease the length of therapy in an effort to reduce the patient's burden, with clinically similar levels of disease control reported.^3^, ^4^, ^5^, ^6^, ^7^ At first progression, re‐irradiation was shown to be beneficial as palliative therapy and has been gaining in popularity.^8^ The efficacy of a second re‐irradiation at second progression has been described in a few cases.^9^ These irradiation approaches are relatively new, with irradiation protocols varying by institution. In this study, we evaluated the efficacy and tolerability of hypofractionated RT compared to conventionally fractionated RT in a single institution, and describe our experience of re‐irradiation and second re‐irradiation.

## Methods

### Patients

We performed a retrospective analysis and review on all patients younger than 18 years of age who were diagnosed with DIPG and received irradiation at Kanagawa Children's Medical Center (KCMC), Japan, between January 2000 and December 2018. This study was conducted after obtaining institutional approval from the Ethics Committee of the KCMC.

A diagnosis of DIPG was based on clinical and imaging characteristics as follows:

- Onset of symptoms ≤3 months before diagnosis.
- Two or more signs of the neurologic triad (cranial nerve defect, ataxia, log tract signs).
- Diffuse infiltration of more than 50% of the axial diameter of the pons, with or without extension to the midbrain and / or medulla oblongata.

Baseline characteristics (age, sex, and the presence of dissemination) were collected from all patients.

### Therapy

All radiation therapy was performed by three‐dimensional conformal radiation (3D CRT) using a linear accelerator. Conventionally fractionated RT was defined as 50.4–59.4 Gy in 28–33 fractions (Fr) of 1.8 Gy daily. In our practice, hypofractionated RT of 44.8 Gy in 16 fractions at 2.8 Gy daily was offered from 2013 as an alternative approach to conventionally fractionated RT. In 2015, we began offering re‐irradiation of 20 Gy in 10 fractions at 2.0 Gy daily at progression to patients with DIPG regrowth as palliative therapy if no other clinical trial was available, since clinical experience with palliative re‐irradiation for recurrent brain tumors had been accumulated in our institution at that time. Written informed consent was obtained from all patients who underwent re‐irradiation and / or their guardians. For re‐irradiation, the area of the recurrent tumor was designated by a gross tumor volume and a minimal margin was added to produce the planned target volume. Information on the use of systemic therapy was collected from patient medical records.

### Assessment

A neurological and general clinical examination performed by an experienced pediatric oncologist before irradiation was made available for all patients. Disease progression was defined as a clinical deterioration in neurological symptoms with the need for steroid use or dose escalation irrespective of confirmation by magnetic resonance imaging (MRI). Clinical re‐evaluation during and after irradiation was performed regularly according to standard practice. Grade 3, 4 and 5 adverse events were registered using the Common Terminology Criteria for Adverse Effects (CTCAE), version 4.0.

### End‐points

The primary end‐point was overall survival (OS), measured from diagnosis to the date of death. Secondary end‐points were safety, based on the frequency of adverse events, and time to progression (TTP), defined as the time to clinical deterioration after an up‐front RT and measured from diagnosis.

### Statistical analysis

All statistical analyses were performed with EZR (Saitama Medical Center, Jichi Medical University, Saitama, Japan), which is a graphical user interface for R (The R Foundation for Statistical Computing, Vienna, Austria). More precisely, it is a modified version of R Commander designed to add statistical functions frequently used in biostatistics.^10^ Fisher's exact test was used to compare patient sex and treatment characteristics. A Mann–Whitney *U*‐test was used to compare patient ages. Overall survival and TTP were calculated with the Kaplan–Meier method, and differences between Kaplan–Meier curves were compared with a log‐rank test.

## Results

### Patient groups and treatment

Twenty‐six children with DIPG were treated between January 2000 and December 2018. Patients who received RT at another institute and whose RT regimen was unknown, and those patients who were under treatment at the time of the analysis, were excluded. The median age at diagnosis was 6.3 years (1.6–14.0 years).

**Figure 1:**
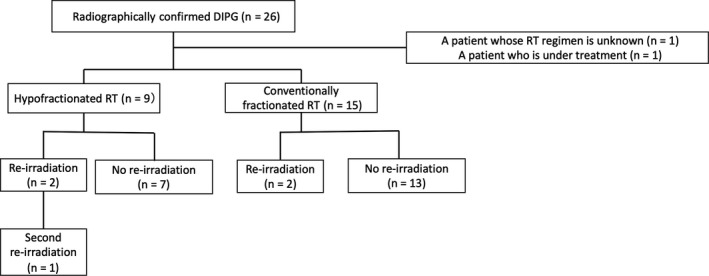
Schematic of patients in our study. Patients were categorized according to treatment. Abbreviations: DIPG, diffuse intrinsic pontine glioma; RT, radiation therapy.

Twenty patients received irradiation only once: 13 patients received conventionally fractionated RT, while seven patients received hypofractionated RT as up‐front RT. Four patients underwent irradiation more than twice (Fig. 1). Patient and treatment characteristics are summarized in Tables 1 and 2.

**Table 1:** The characteristics of patients who received radiation therapy only once

Characteristic	Conventionally fractionated RT (*n *= 13)	Hypofractionated RT (*n *= 7)	*P*
Gender			
Male	6	5	
Female	7	2	0.37
Age (years)			
Median	6	7	
Range	1‐9	4‐14	0.87
Treatment			
No CT	9	1	
CT	4	6	0.06
TTP (months)	4.2 (95%CI: 1.8–8.3)	7.1 (95%CI: 4.5–8.7)	0.38
OS (months)	9.7 (95%CI: 7.1–11.2)	11.0 (95%CI: 5.2–13.6)	0.6

Abbreviations: CT, chemotherapy; OS, overall survival; RT, radiation therapy; TTP, time to progression.

**Table 2:** The characteristics and clinical courses of four patients who received re‐irradiation

Patient	Age （years)	Sex	Treatment at diagnosis	Time to first progression (days)	Treatment at first progression	Time to second RT (days)	Time to second progression (days)	Time to third RT (days)	OS (days)
1	9	F	conventionally fractionated RT + TMZ	243	BV → second RT + BV	305	372	‐	460
2	4	M	hypofractionated RT	233	second RT	258	396	426	558
3	9	F	conventionally fractionated RT	208	TMZ → second RT	258	281	‐	395
4	5	F	hypofractionated RT + BV	213	second RT + BV	231	276	‐	415

Abbreviations: BV, bevacizumab, OS, overall survival; RT, radiation therapy; TMZ, temozolomide.

### Hypofractionated RT

**Figure 2:**
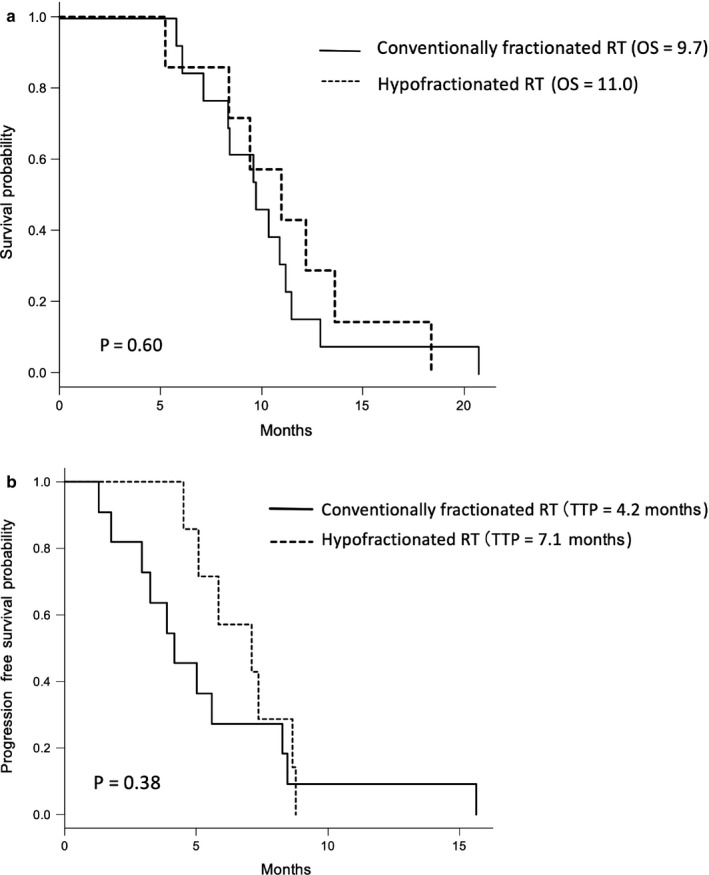
Kaplan–Meier estimates of overall survival (OS) from initial diagnosis (a) and time to progression (TTP) (b) of conventionally fractionated radiotherapy and hypofractionated radiotherapy groups. *P*‐value derived from log‐rank test across cohorts. Abbreviations: OS, overall survival; TTP, time to progression.

Excluding patients who underwent re‐irradiation, a statistically significant difference in age at diagnosis (*P *= 0.87) or sex distribution (*P *= 0.37) was not noted between patients who received hypofractionated RT and those who received conventionally fractionated RT. Patients in the hypofractionated RT group more commonly received systemic chemotherapy in addition to radiotherapy: six of seven (86%) patients in the hypofractionated RT group versus 9 out of 13 (69%) in the conventionally fractionated RT group. A statistically significant difference was not observed (*P *= 0.057). The median OS of the hypofractionated RT group was 11.0 months (95% CI, 5.2–13.6 months), which is similar to that of the conventionally fractionated RT group (9.7 months [95% CI:7.1–11.2], *P *= 0.60; Table 1). The OS at 6, 9, and 12 months was 86%, 71%, and 29%, respectively, in the hypofractionated RT group, and 92%, 62%, and 15.4%, respectively in the conventionally fractionated RT group (Fig. 2a).

No significant difference in TTP was observed between hypofractionated and conventionally fractionated RT groups: 7.1 months (95％ CI: 4.5–8.7 months) versus 4.2 months (95％ CI: 1.8–8.3 months), respectively (*P *= 0.38; Fig. 2b).

No grade 3 or 4 toxicities were observed in patients who received hypofractionated RT.

### Re‐irradiation

Four patients underwent re‐irradiation at first progression. The details of treatment and clinical courses are summarized in Table 2. All patients showed a transient neurological improvement and lived more than a year after diagnosis, which was longer than the median OS of patients who did not undergo re‐irradiation (10.0 months [95％ CI: 8.3–11.5 months]).

### A case of second re‐irradiation

A 4‐year‐old boy presented with a 2 week history of headaches, articulation disorder, and ataxia. A brain MRI showed a large pontine T1 hypointense/T2 hyperintense mass with contrast ring enhancement, which was compatible with DIPG. Hypofractionated RT (44.8 Gy/16 Fr) was administered with good tolerance and a subsequent improvement in neurological symptoms. Post‐RT imaging showed a decrease in tumor bulk within the pons. No post‐RT systemic therapy was offered and the patient remained well for 7.7 months. Thereafter, he presented with dysbasia and a subsequent MRI revealed tumor progression. Re‐irradiation with 20 Gy/10 Fr was administered with good tolerance. An improvement in neurological symptoms was observed 3 days after the initiation of re‐irradiation, with the patient able to run and jump by himself after one month. Four months later, the patient's condition deteriorated again and a second course of focal re‐irradiation with 20 Gy/10 Fr was administered 5‐and‐a‐half months after the first re‐irradiation. The patient regained transient neurological improvement and remained well for 2 months. He died of tumor progression 5 months after the second re‐irradiation. An autopsy was performed and a diagnosis of a diffuse midline glioma with a H3.3 K27M mutation was made. Histologically, degenerative changes associated with radiation were limited, with tumor cells in most areas viable. Mitotic features were rare, and the Ki‐67 labeling index was less than 5%.

## Discussion

Radiation therapy for DIPG is considered aggressive palliative therapy because it prolongs survival by a mean of 3–6 months but is not curative.^11^ For this life‐limiting condition, improving the quality of remaining life while decreasing the burden of treatment is strongly desired.

Hypofractionated RT has the advantage of the completion of treatment in about 3 weeks instead of the 6 weeks of conventionally fractionated RT. For young children, in particular, who require anesthesia for irradiation, any reduction in the duration of therapy directly improves their quality of life by reducing any anesthesia requirements, such as diet restrictions. Janssens *et al. *described how a hypofractionated regimen (39 Gy/13 Fr or 44.8 Gy/16 Fr) offered equal OS with a decreased treatment burden compared with a conventional regimen in a matched‐cohort analysis.^3^ A phase Ⅲ randomized trial of hypofractionated RT for DIPG did not reveal the generally assumed non‐inferiority of conventionally fractionated RT, with a median progression‐free survival of 6.3 months in the hypofractionated arm versus 7.3 months in the conventionally fractionated arm. However, hypofractionated RT was well tolerated and the survival between both arms was nearly comparable.^7^ Our study is limited by its retrospective design and the small cohort used but supports the observation that the TTP and OS of patients with DIPG treated with hypofractionated RT were clinically similar to those of patients treated with conventionally fractionated RT. Although the risk of an adverse effect from RT on adjacent normal tissue was increased, hypofractionated RT was well tolerated in our study. In view of the role of RT in DIPG as a palliative intervention, it may be preferable for the method of front‐line RT to be determined on a case‐by‐case basis because of considerations such as the patient's quality of life and the potential risk of earlier disease re‐progression after hypofractionated RT.

At first progression, evidence about the feasibility of palliative re‐irradiation in DIPG is accumulated. Some institutions provide palliative re‐irradiation in the absence of effective treatment options for recurrent DIPG and report tolerability and durable tumor control.^11^, ^12^ A multicenter retrospective matched‐cohort analysis conducted by the European Society of Pediatric Oncology high‐grade glioma (SIOP‐E‐HGG)/DIPG working group showed a benefit in OS (13.7 versus 10.3 months; *P *= 0.04) of re‐irradiation at first progression for patients with DIPG responding to up‐front radiotherapy. In this study, a clinical improvement with re‐irradiation was observed in 24/31 (77%) patients and no grade 4–5 toxicities were recorded.^8^ The re‐irradiation regimen varied between institutions: e.g. 18.0–21.6 Gy in 1.8–2.0 Gy fractions, 30 Gy in 3.0 Gy fractions, or 30.6–36 Gy in 1.8 Gy fractions. A Phase Ⅰ/Ⅱ trial suggested that a regimen of 24 Gy in 2.0 Gy fractions was preferred;^13^ however, further research is warranted to optimize the dose. We provided re‐irradiation of 20 Gy in 2.0 Gy fractions at first progression to four patients. All patients showed a clinical improvement and comparatively longer OS to the previously reported median OS of DIPG. The time between the start of the second RT and second progression varied from 23 to 138 days. These findings should be carefully applied in clinical situations because of a possible selection bias introduced by the retrospective nature of these studies on the re‐irradiation of DIPG.

The patient with the longest disease‐controlled period after re‐irradiation underwent a second re‐irradiation at second progression, based on a previously reported second re‐irradiation regimen of 20 Gy/10 Fr.^9^ He again showed a clinical improvement that lasted 2 months. An autopsy revealed a H3.3 K27M mutation, which was associated with a longer median OS,^14^ and low mitotic features in the tumor. This may explain the patient's good response to re‐irradiation and a second re‐irradiation. It therefore follows that understanding the clinical and biological features of a good responder to RT will aid in the selection of patients who are expected to gain benefits from re‐irradiation and a second re‐irradiation.

In conclusion, our study indicated that up‐front hypofractionated RT can reduce the burden of treatment in children with DIPG without jeopardizing OS and TTP. Re‐irradiation at first progression is suggested to be beneficial.

## Disclosure

The authors declare no conflict of interest.

## Author contributions

A.H. designed the study, conducted the initial analysis, and drafted the initial manuscript. H.G. conceptualized the study and revised the manuscript. O.M., I.E., A.N., M.T., Y.T., and H.S. reviewed and revised the manuscript. N.T., F.I., S.H., and T.Y. were responsible for the evaluation of patients. All authors read and approved the final manuscript.

## References

[1] Jackson S. , Patay Z. , Howarth R. *et al* Clinico‐radiologic characteristics of long‐term survivors of diffuse intrinsic pontine glioma. J. Neurooncol. 2013; 114: 339–44. 23813229 10.1007/s11060-013-1189-0 PMC3755743

[2] Cohen K.J. , Jabado N. , Grill J. . Diffuse intrinsic pontine gliomas ‐ Current management and new biologic insights. Is there a glimmer of hope? Neuro. Oncol. 2017; 19: 1025–34. 28371920 10.1093/neuonc/nox021 PMC5570259

[3] Jannsens G.O. , Jannsen M.H. , Lauwers S.J. *et al* Hypofractionation vs conventional radiation therapy for newly diagnosed diffuse intrinsic pontine glioma: A matched‐cohort analysis. Int. J. Radiat. Oncol. Biol. Phys. 2013; 85: 315–20. 22682807 10.1016/j.ijrobp.2012.04.006

[4] Negretti L. , Bouchireb K. , Levy‐Piedbois C. *et al* Hypofractionated radiotherapy in the treatment of diffuse intrinsic pontine glioma in children: A single institution's experience. J. Neurooncol. 2011; 104: 773–7. 21327862 10.1007/s11060-011-0542-4

[5] Zaghloul M.S. . Has hypofractionated radiotherapy become the standard of care in pediatric DIPG? Child's Nerv. Syst. 2015; 31: 1221–2. 25761846 10.1007/s00381-015-2678-6

[6] Hankinson C.T. , Patibandla M.R. , Green A. *et al* Hypofractionated radiotherapy for children with diffuse intrinsic pontine gliomas. Pediatr. Blood Cancer. 2016; 63: 716–8. 26544789 10.1002/pbc.25836

[7] Zaghloul M.S. , Eldebawy E. , Ahmed S. *et al* Hypofractionated conformal radiotherapy for pediatric diffuse intrinsic pontine glioma (DIPG): A randomized controlled trial. Radiother. Oncol. 2014; 111: 35–40. 24560760 10.1016/j.radonc.2014.01.013

[8] Janssens G.O. , Gandola L. , Bolle S. *et al* Survival benefit for patients with diffuse intrinsic pontine glioma (DIPG) undergoing re‐irradiation at first progression: A matched‐cohort analysis on behalf of the SIOP‐E‐HGG/DIPG working group. Eur. J. Cancer. 2017; 73: 38–47. 28161497 10.1016/j.ejca.2016.12.007

[9] La Madrid A.M. , Santa‐María V. , Cruz Martinez O. *et al* Second re‐irradiation for DIPG progression, re‐considering “old strategies” with new approaches. Child's Nerv. Syst. 2017; 33: 849–52. 28251325 10.1007/s00381-017-3352-y

[10] Kanda Y. . Investigation of the freely available easy‐to‐use software “EZR” for medical statistics. Bone Marrow Transplant. 2013; 48: 452–8. 23208313 10.1038/bmt.2012.244 PMC3590441

[11] Vanan M.I. , Eisenstat D.D. . DIPG in children – what can we learn from the past? Front. Oncol. 2015; 5: 237. 26557503 10.3389/fonc.2015.00237 PMC4617108

[12] Freese C. , Takiar V. , Fouladi M. , DeWire M. , Breneman J. , Pater L. . Radiation and subsequent reirradiation outcomes in the treatment of diffuse intrinsic pontine glioma and a systematic review of the reirradiation literature. Pract. Radiat. Oncol. 2017; 7: 86–92. 28274399 10.1016/j.prro.2016.11.005

[13] Amsbaugh M.J. , Mahajan A. , Thall P.F. *et al* A phase I/II trial of reirradiation for diffuse intrinsic pontine glioma. Int. J. Radiat. Oncol. Biol. Phys. 2019; 104: 144–48. 30610915 10.1016/j.ijrobp.2018.12.043

[14] Hoffman L.M. , Veldhuijzen Van Zanten SEM , Colditz N. *et al* pathologic, and molecular characteristics of long‐term survivors of Diffuse Intrinsic Pontine Glioma (DIPG): A collaborative report from the International and European Society for Pediatric Oncology DIPG registries. J. Clin. Oncol. 2018; 36: 1963–72. 29746225 10.1200/JCO.2017.75.9308 PMC6075859

